# A classification system for identifying patients dead on ambulance arrival: a prehospital medical record review

**DOI:** 10.1186/s13049-023-01171-0

**Published:** 2023-12-21

**Authors:** Markus Petersen, Fredderick Georg Kjeldtoft, Erika Frischknecht Christensen, Henrik Bøggild, Tim Alex Lindskou

**Affiliations:** 1 Centre for Prehospital and Emergency Research, Aalborg University Hospital and Department of Clinical Medicine, Aalborg University, Gistrup, Denmark; 2 Department of Emergency and Trauma Care, Clinic of Internal and Emergency Medicine, Aalborg, Denmark; 3 Public Health and Epidemiology, Department of Health Science and Technology, Aalborg University, Gistrup, Denmark; 4 Clinical Biostatistics, Aalborg University Hospital, Aalborg, Denmark

**Keywords:** Prehospital, Mortality, Emergency medical services, Dead on ambulance arrival, Ambulance, Out-of-hospital cardiac arrest

## Abstract

**Background:**

Patients dead before arrival of the ambulance or before arrival at hospital may be in- or excluded in mortality analyses, making comparison of mortality difficult. Often only physicians are allowed to declare death, thereby impeding uniform registration of prehospital death. Many studies do not report detailed definitions of prehospital mortality. Our aim was to define criteria to identify and categorize prehospital patients’ vital status, and to estimate the proportion of these groups, primarily the proportion of patients dead on ambulance arrival.

**Methods:**

Prehospital medical records review for patients receiving an ambulance in the North Denmark Region from 2019 to 2021 and registered dead on the same or the following day. We defined three vital status categories: (1) Dead on Ambulance Arrival (DOAA), (2) Out-of-Hospital Cardiac Arrest (OHCA) divided into OHCA Basic Life Support (OHCA BLS) and OHCA Advanced treatment, and 3) Alive on Ambulance Arrival.

**Results:**

Among 3 174 dead patients, DOAA constituted 28.8%, OHCA BLS 13.4%, OHCA Advanced treatment 31.3%, and Alive on Ambulance Arrival 26.6%.

**Conclusion:**

We defined exhaustive and mutually exclusive criteria to define vital status, DOAA, OHCA, and Alive on Ambulance Arrival based on prehospital medical records. More than one out of four patients receiving an ambulance and registered dead on the same or the following day were dead already at ambulance arrival. Adding OHCA BLS where resuscitation was terminated without defibrillation or other treatment, increased the proportion of patients dead on ambulance arrival to 42%. We recommend reporting similar categories of vital status to improve valid comparisons of prehospital mortality rates.

## Background

Mortality is a key parameter to assess patient outcome and often used as a quality indicator in cardiac arrest and trauma registries, as well as in general emergency and prehospital studies [1–7]. Measurement of mortality presents a challenge in emergency patients, both in the prehospital field and in the emergency department, as patients may be either perceived or clinically dead before arrival of an ambulance on the scene, or before arrival at a hospital. This key issue has been addressed differently with variations in categorization of the prehospital deaths and by in- or excluding these in mortality calculations. In the prehospital setting, uniform reporting of death is hampered by differences in legislation concerning, amongst others, declaration and registration of death on the scene and rules for initiation and termination of resuscitation [8–12]. Studies’ escriptions of these issues are scarce, and there is currently not a uniform definition and no consensus on reporting of prehospital deaths [13–17]. The lack of uniform definitions and registrations of death on the scene impedes the comparisons of survival rates in emergency patient populations, in research as well as in clinical quality registries such as in trauma and out-of-hospital cardiac arrest registries [5–7, 18].

In Denmark a physician is required to declare a patient dead [8]. Paramedics are, except in certain situations (extensive decay or obviously fatal injuries incompatible with continued life), not allowed to declare a patient dead and must initiate or continue treatment until contact with a physician. As such, patients without the potential for resuscitation may receive treatment and are registered as cardiac arrest patients even if they would have been declared dead if a physician had been present. In continuation hereof, death may be declared and registered after arrival at a hospital, rather than in the prehospital setting [19, 20]. Altogether this may influence on the reported survival and mortality rates and call upon uniform definitions.

In Denmark, the emergency medical services use a prehospital medical record, but complete and correct registration of prehospital death is not possible due to the legislative issues mentioned above. Review of the full prehospital medical record can provide the needed insight regarding the vital status of the patient during the prehospital care phase, as the records contain patient information, vital signs, observations, treatment etc.

As such, our aim was to develop simple and reproducible criteria to identify and categorize prehospital patients’ vital status (i.e. dead or cardiac arrest), and based on this to estimate the proportion of prehospital deaths, primarily the proportion of patients dead on ambulance arrival.

## Methods

### Design

Cross sectional study of patients deceased after calling for an ambulance in the North Denmark Region with review of their prehospital medical records to assess the patients’ vital status at ambulance arrival.

### Setting

Denmark has a population of 5.9 million, of which approximately 10% live in the North Denmark Region [21]. The Danish healthcare system is tax-financed and free to all residents [22]. Residents are assigned a unique civil registration number, which holds information about age and sex and is a personal identifier in national registries and medical records.

The Danish emergency medical service is regionally organized and consists of several dispatch options, including lay-bystander first responders, paramedics, and physicians (anesthesiologists) [23].

The prehospital medical record contains information on initial emergency call, dispatch information, civil registration number, observations, automatically transferred vital signs from a monitor/defibrillator, drugs, treatment, cardiac arrest, and note fields [23].

### Declaration of death

In Denmark, the declaration of death requires a physician unless the person is found with lay-bystander signs of death (extensive decay or obviously fatal injuries incompatible with continued life), or the death was assessed as imminent by a physician directly involved in the care of the patient in the time before death. [8] Bystanders must, to the best of their abilities, help distressed individuals, whereas health care professionals, including paramedics, are obliged to initiate or continue cardiopulmonary resuscitation (CPR) until a physician takes over or terminates the treatment. A physician issues a death certificate when at least one of the late signs of death, stiffness of death (rigor mortis), postmortem lividity (livores mortis), and putrefaction (cadaverositas), are present. This can, depending on the situation, be performed on the scene or at the emergency department. The dead body may be left on the scene or taken to a local hospital or morgue until burial or cremation [8, 24]. After death, the citizens are registered as dead in the Danish Civil Registration System [25].

### Study population

We included patients deceased on the same or following day after calling the national emergency number, 112, requesting an ambulance in the North Denmark Region in the period January 2019 to December 2021. Only patients with a valid Civil Registration Number, and who were registered as dead in the Danish Civil Registration System, were included.

### Data sources

The prehospital medical record was the primary data source. The Danish Civil Registration System provided information on date of death [26]. To secure inclusion of all deaths within the first 24 h after the emergency call, we included patients dead on the same or the following date as receiving an ambulance because the registry does not contain the exact time, only the date of death. This means that the included patients all deceased within a maximum of 48 h after the emergency call, either in the prehospital phase or after, at either hospital or at home. The datasets were linked using the patients’ unique civil registration numbers.

### Prehospital medical record review and definitions

The medical review was an iterative process where the authors through discussions developed exhaustive and mutually exclusive categories of vital status at ambulance arrival and during the prehospital phase. The two first-authors defined the initial set of criteria for categorizing the patients’ vital status by performing a complete evaluation of a random sample of 100 prehospital medical records. The evaluation included the reason for calling an emergency, dispatch information, vital signs, drugs administered, treatment given, cardiac arrest section (where applicable), and note fields for the entire prehospital medical record. This was discussed by the entire author group and refined and redesigned until the final exhaustive and mutually exclusive categories were reached: 1) Dead on Ambulance Arrival (DOAA), 2) Out-of-Hospital Cardiac Arrest (OHCA), 3) Alive on Ambulance Arrival, and 4) No clear category. Table 1 describes these final categories and definitions in details, applicable for others to use. After the process of developing the categories, the two first authors assessed inter-rater reliability in a new random sample of 100 prehospital medical records. Finally, they reviewed all prehospital medical records individually and in case the patient’s vital status was unclear, discussed with senior authors.

**Table 1:** Categories. Definitions and specific criteria for vital status category based on prehospital medical records

**Dead on Ambulance Arrival (DOAA)**
•*Patient found with extensive decay or obviously fatal injuries incompatible with continued life (*= *lay-bystander signs of death)*
or
•*Described in the note fields that the patient was found with no signs of life and no vital signs registered or vital signs* = *0, no resuscitation attempt, no treatment, and no drugs administered*
**Out-of-hospital Cardiac Arrest (OHCA)**
OHCA was defined according to the Utstein Guidelines [27] as patients where resuscitation was initiated. Patients found with, or developing, OHCA were sub-categorized according to the level of treatment:
Basic Life Support only (OHCA BLS)
•*Patient only received basic life support (manual chest compressions and/or rescue breaths, NO defibrillation) – either by bystander only or by any emergency healthcare professional*
Advanced treatment (OHCA Advanced treatment)
• *Patient received basic life support and defibrillation and/or any advanced treatment (intubation, fluid therapy, adrenalin, mechanical chest compression, oxygen therapy, airway management equipment, or other)*
**Alive on Ambulance Arrival**
Died after prehospital care
• *Patients released alive on the scene or brought to a hospital alive without cardiac arrest at any point*
End-of-life care
• *Patient received only end-of-life care and died on the scene or was released on the scene to end-of-life care, no resuscitation attempted*
**No clear category**
• *Prehospital medical record incomplete*
or
• *Ambulance mission cancelled before arrival at the patient*

**Fig. 1:**
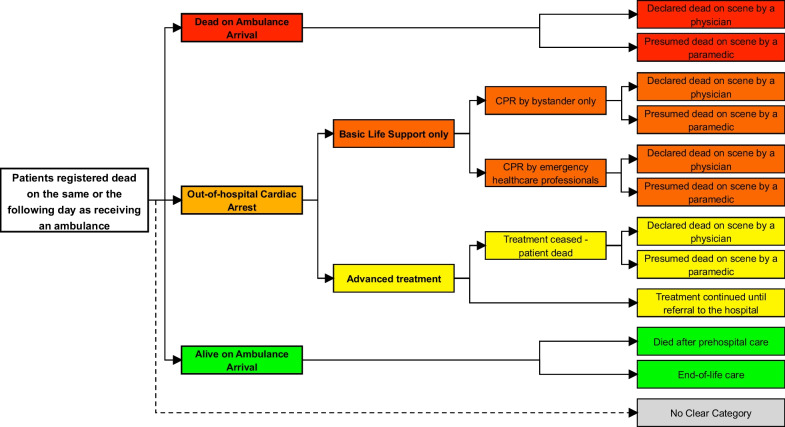
Vital status categories and outcomes. Overview of the categorization and subcategories of vital status. CPR, cardiopulmonary resuscitation

Patients were further classified as *declared dead* by a physician on the scene, or *presumed dead* by a paramedic after conferring with a physician who gave permission not to initiate CPR, or to terminate ongoing CPR in case of OHCA (Fig. 1).

### Statistical analysis

Patient data were entered and managed using REDCap electronic data capture tools hosted at the North Denmark Region. [28, 29] The “No clear category” was calculated but excluded from the analyses.

We presented descriptive statistics as proportion, median and interquartile range (IQR), and for comparative analyses we used Pearson Chi-square and Mann–Whitney U test. We stratified on year of the study period. Cohen’s Kappa was used to measure inter-rater reliability between the two authors, estimated with a 95% confidence interval.

Comparisons with a *p*-value < 0.05 were considered statistically significant.

Statistical analyses were conducted in StataCorp. 2021. Stata Statistical Software: Release 17. College Station, TX: StataCorp LLC with the plugin “kappaetc” (Daniel Klein, Universität Kassel).

## Results

**Fig. 2:**
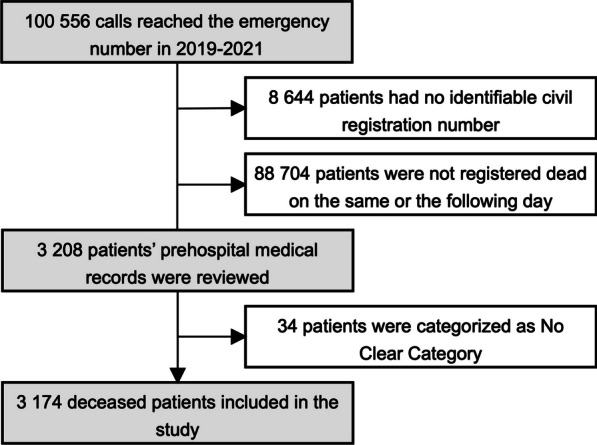
Flowchart Inclusion of deceased patients who received an ambulance after calling the emergency number in 2019–2021 in the North Denmark Region and were registered dead on the same or the following day

In 2019–2021 there were 100 556 calls to the emergency number in the North Denmark Region. A total of 3 208 patients received an ambulance and were registered dead in the Danish Civil Registration System on the same or the following date. A small fraction, 1.1% of patients (n = 34) were categorized as “No clear category” and excluded. Thus, we included 3 174 deceased patients (Fig. 2).

All included patients’ vital status could be categorized, of which 30 (0.94%) cases were settled by discussion. The first and second authors agreed in 96/100 of the cases in the inter-rater reliability test, Cohen’s Kappa was 0.95 (95% confidence interval: 0.90–0.99). Most cases of disagreement concerned whether DOAA patients were declared dead by a physician or presumed dead by a paramedic.

**Fig. 3:**
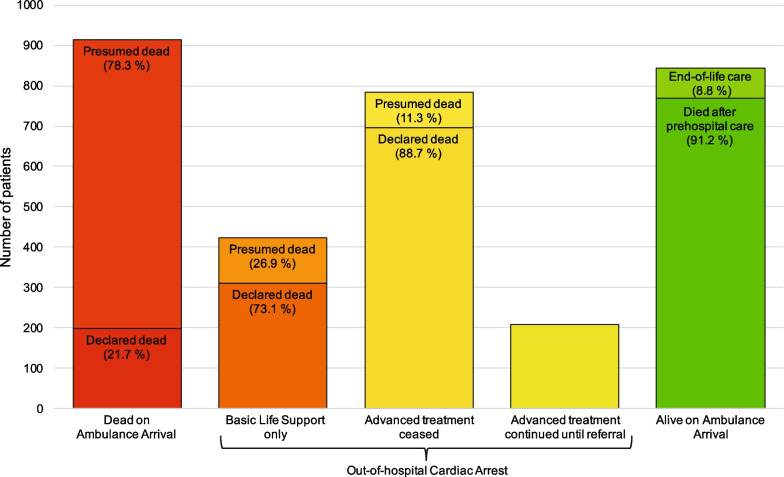
Patients presumed and declared dead Vital status category in 3 174 ambulance patients registered dead in the Danish Civil Registration System on the same day or the day after the emergency call during the years 2019–2021 showing proportions of deaths presumed by paramedics and declared by physicians on the scene

Patients assessed as DOAA accounted for 28.8%. OHCA patients in total accounted for 44.6%, with 13.4% only receiving basic life support (only manual chest compression and rescue breaths and no defibrillation). OHCA with advanced treatment was given in 31.3%, and in 6.6% advanced treatment continued until handover at a hospital. Finally, 24.2% were assessed as Died after prehospital care and 2.3% received end-of-life care (Table 2 and Fig. 3).

**Table 2:** Vital status category. Vital status category in 3 174 ambulance patients dead the same day or the day after the emergency call during the years 2019–2021. CPR, cardiopulmonary resuscitation

	2019 n (%)	2020 n (%)	2021 n (%)	Total n (%)
Total	1 030 (32.5%)	1 018 (32.1%)	1 126 (35.5%)	3 174 (100%)
**Dead on Ambulance Arrival**	300 (29.1%)	285 (28.0%)	329 (29.2%)	914 (28.8%)
**Out-of-hospital Cardiac Arrest**	452 (43.8%)	476 (46.8%)	489 (43.4%)	1 417 (44.6%)
Basic Life Support only	125 (12.1%)	139 (13.7%)	160 (14.2%)	424 (13.4%)
CPR by bystander only	51 (5.0%)	60 (5.9%)	71 (6.4%)	182 (5.7%)
CPR by emergency healthcare professional	74 (7.2%)	79 (7.8%)	89 (7.9%)	242 (7.6%)
Advanced treatment	327 (31.7%)	337 (33.1%)	329 (29.2%)	993 (31.3%)
Treatment terminated	262 (25.4%)	265 (26.0%)	258 (22.9%)	785 (24.7%)
Treatment continued	65 (6.3%)	72 (7.1%)	71 (6.3%)	208 (6.6%)
**Alive on Ambulance Arrival**	278 (27.0%)	257 (25.2%)	308 (27.4%)	843 (26.6%)
Died after prehospital care	258 (25.0%)	236 (23.2%)	275 (24.4%)	769 (24.2%)
End-of-life care	20 (1.9%)	21 (2.1%)	33 (2.9%)	74 (2.3%)

There was no statistically significant difference in the distribution of the categories over the period (Pearson Chi-square, *p* = 0.180).

Overall, males constituted most of the patients (61.8%) (*p* < 0.00049) and were younger (median 73, IQR 63–81) compared to females (median 77, IQR 67–85) (*p* = 0.0001) (Table 3).

**Table 3:** Vital status category and characteristics. Age, sex, and vital status category at ambulance arrival in 3 174 patients registered dead on the same day or the day after the emergency call during the years 2019–2021. CPR, cardiopulmonary resuscitation

	N	%	Sex % female	Age, median, IQR (years)
**Dead on Ambulance Arrival**	**914**	**28.8**	**34.1**	**All: 71 (60–80), F: 74 (64–84), M: 69 (58–77)**
Declared dead	198	6.2		
Presumed dead	716	22.6		
**Out-of-hospital Cardiac Arrest**	**1417**	**44.6**	**36.5**	**All: 73 (63–81), F: 76 (65–83), M: 72 (61–79)**
Basic Life Support only	424	13.4	38.9	All: 75.5 (65–83), F: 79 (66–85), M: 74 (64–81)
*CPR by bystander only*				
Declared dead	125	3.9		
Presumed dead	57	1.8		
*CPR by emergency healthcare professional*				
Declared dead	185	5.8		
Presumed dead	57	1.8		
Advanced treatment	993	31.3	35.5	All: 72 (61–80), F: 74 (64–81), M: 71 (60–79)
*Treatment terminated*				
Declared dead	696	21.9		
Presumed dead	89	2.8		
*Treatment continued*	208	6.6		
**Alive on Ambulance Arrival**	**843**	**26.6**	**45.3**	**All: 80 (72–86), F: 81.5 (74–87), M: 78 (71–85)**
Died after prehospital care	769	24.2		
End-of-life care	74	2.3		

Males constituted the majority of both DOAA and OHCA BLS patients (65.9% and 61.1%) and were younger than females (Table 3), and OHCA BLS patients were significantly older at death than the DOAA patients (*p* = 0.0001). DOAA patients were mainly presumed dead by a paramedic (78.3%), whereas a physician most often declared the OHCA patients dead (Fig. 3 and Table 3).

## Discussion

### Principal findings

In this cross-sectional review of medical record of more than 3 000 patients receiving an ambulance after calling the national emergency number and registered dead on the same or the following day in the Danish Civil Registration System, more than one in four were already dead when the ambulance arrived.

Patients were classified as OHCA BLS (manual chest compressions and rescue breaths only) even when BLS was given for a short time as recommended in the Utstein guidelines for defining OHCA [27]. As no defibrillator was used nor any drugs administered or other advanced treatment and resuscitation continued, the CPR was most likely initiated by bystanders and/or paramedics as obligated by law and continued until terminated by a physician. The OHCA BLS only patients thus may be considered as futile for resuscitation. Adding these to the DOAA may provide a better estimate of patient dead at ambulance arrival. The patients who were classified as having died after prehospital care, are a particularly interesting group, accounting for a fourth of all included patients. Due to the inclusion criteria, a portion of these patients may have died the following day of the ambulance run. This makes them a possible highly acute group, and further exploring these patients may identify meaningful early intervention possibilities.

### Strengths and limitations

An important strength of this study of patients to whom an ambulance was dispatched was its population-based design and the tax-financed healthcare system in Denmark, ensuring free prehospital service to all citizens which reduced selection bias. Another strength was the data linkage due to the civil registration number and the small fraction of patients (8.5%) with missing civil registration number, compared to previous studies of emergency medical services [2, 3, 30]. The Danish Civil Registration System provided information on vital status and ensured a virtually complete information on death [26].

We included three years of data to obtain a large sample size and make the study robust against temporal variation. The thorough reviewing of the entire prehospital medical record and the strong inter-rater agreement elevated the study’s internal validity [31]. The categories were simple and developed as mutually exclusive and exhaustive. The number of cases difficult to categorize was low as a joint review was needed in only 30 out of all cases (0.94%).

Although we only included data from one region in Denmark, we anticipate that the uniform legislation and organization would imply that the results are relevant for the entire country. However, our specific results cannot be generalized to all emergency medical systems as it depends on the legislation concerning pronouncing death prehospitally, rules of termination, the availability of prehospital physicians etc. The vital status categories were developed and defined to be simple and applicable in other settings with prehospital medical records.

### Comparison with other studies

Prehospital and in-hospital studies have investigated the proportion of patients dead in the prehospital setting, but with different definitions of prehospital death [13, 15–17]. A Norwegian study by Bakke et al. explored the impact of rurality on the epidemiology of prehospital trauma patients [13]. They reviewed medical records, and to examine when the patients died, they defined death as “the point in time where the patient became lifeless and no attempt at resuscitation was made or such attempts were terminated. As such, death did not require a physician to declare the patient dead [13].” Mills et al. examined the association between time from highest priority emergency medical service vehicle dispatch to hospital arrival and 1-day and 30-day mortality in a Danish population [16]. They used the term declared dead in the field, defined as “obvious signs of death (ie livores and/or rigor mortis) or the prehospital emergency physician was present according to the Danish legislation” [16]

In the United States, Handberry et al. who assessed the changes in emergency medical service activations before and during the COVID-19 pandemic and Mueller et al. who described the characteristics of emergency medical service care response in frontier and remote areas, defined on the scene death as dead on emergency medical service arrival or dead after arrival with unsuccessful field resuscitation [15, 17]. This definition seems like a combination of our categories DOAA, OHCA BLS and OHCA Advanced treatment terminated. It is not stated how, and by who, the patients in the studies by Handberry et al. and Mueller et al. were declared and registered dead on the scene.

International comparisons of OHCA survival rates such as by the ILCOR-group are also affected by the inclusion criteria and definition of OHCA versus dead on ambulance arrival, and the different countries’ criteria for the latter are not reported [7]. In the National Danish Cardiac Arrest Registry exclusion criteria are “cases with late signs of death (decomposition, rigor mortis, or livor mortis) or lesions incompatible with sustained life” [18].

The variations across studies in definitions of prehospital death impede comparability and may result in underestimation as well as overestimation of mortality/survival.

### Clinical implications and future perspectives

We suggest defining specific criteria when describing and reporting the proportion of patients dead on ambulance arrival or in the prehospital phase to improve comparability of mortality among emergency patients in general as well as among selected groups such as out-of-hospital cardiac arrest. Using the definitions presented in this study eases comparison across studies and clinical quality registries. The vital status categories may also serve as criteria for selecting cases for medical audit in local clinical quality control and improvement.

Our study was based on reviews of detailed prehospital medical records, which was time-consuming, but our developed categories are simple and applicable in other settings where paramedics alone cannot legally declare patient dead. Furthermore, these categories constitute a basic ‘golden standard’ material for future research developing algorithms and/or text-mining protocols to identify prehospital deaths.

## Conclusion

In a review of more than 3 000 prehospital medical records on ambulance patients registered dead on the same or the following day, development of three categories of vital status on the scene made it possible to estimate the proportion of prehospital deaths. In our setting, 25% of the patients were already dead when the ambulance arrived on the scene, raising the proportion to 40% when adding futile resuscitations given only compressions/ventilations. Thus, patients dead on ambulance arrival may constitute a considerable proportion of all deaths within the first day after receiving an ambulance. As the legislation about declaring death on the scene differs between countries, uniform definitions and registrations of prehospital death are essential to ensure high-quality statistics when reporting mortality in emergency and prehospital studies and quality registers.

## Data Availability

The data that support the findings they are not publicly available, and restrictions apply. Upon reasonable request and with permission and approval for the handover of patient medical records from the North Denmark Region, data are available and may be requested from the North Denmark Region Emergency Medical Services (DenPraehospitaleVirksomhed@rn.dk).

## Abbreviations

OHCA: Out-of-Hospital Cardiac Arrest
DOAA: Dead on Ambulance Arrival
CPR: Cardiopulmonary resuscitation
BLS: Basic Life Support
IQR: Interquartile range
